# Impact of Diverse Clinical Characteristics on Survival Benefit of Liver Transplantation

**DOI:** 10.1155/joot/1291289

**Published:** 2026-01-15

**Authors:** Mouaid Alim, Bishoy Lawendy, Shiyi Chen, Naomi Khaing Than Hlaing, Saba Maleki, Mamatha Bhat

**Affiliations:** ^1^ Ajmera Transplant Program, University Health Network, Toronto, Ontario, Canada, uhn.ca; ^2^ Department of Computer Science, University of Toronto, Toronto, Ontario, Canada, utoronto.ca; ^3^ Department of Gastroenterology, Western University, London, Ontario, Canada, uwo.ca; ^4^ Division of Gastroenterology, University of Toronto Faculty of Medicine, Toronto, Ontario, Canada, utoronto.ca; ^5^ Toronto General Hospital Research Institute, University Health Network, Toronto, Ontario, Canada, uhn.ca

**Keywords:** clinical characteristics, demographic features, liver transplant, MELD score, survival benefit

## Abstract

**Background:**

Liver transplantation (LT) is offered as a life‐saving treatment to those with end‐stage liver disease. There has recently been much interest in considering the survival benefit of transplant when prioritizing patients for transplant. In this study, we aimed to measure the survival benefit of LT across various patient demographics by measuring the number of life‐years gained after LT.

**Method:**

In this study, 101,770 patients were included from the Scientific Registry of Transplant Recipients (SRTR) transplanted between 2003 and 2021. The survival benefit of LT was calculated using restricted mean survival time (RMST) in the next 16 years post‐transplant. Cox proportional hazard models and Weibull models were employed to quantify the impact of various predictors on survival benefit.

**Results:**

LT was found to provide survival gains of 4.93, 6.43, 6.30, and 3.67 years for the 18–29, 30–49, 50–69, and 70+ age groups, respectively. Survival benefit was highest at 6.62 years for patients with model for end‐stage liver disease (MELD) scores of 15–19, with survival benefits of 5.7, 5.4, and 5.85 years for the 6–14, 20–29, and 30–40 MELD score cohorts, respectively. Older age (HR: 1.13), male sex (HR: 1.32), diabetes (HR: 1.32), and higher MELD scores were predictors of lesser survival benefit. Protective factors included higher education levels (HR: 0.70) as well as diagnoses of fulminant liver failure and autoimmune biliary disease (HR: 0.65).

**Conclusion:**

This study underscores how survival benefit varies across patients with different demographic and clinical characteristics, highlighting the nuanced interplay of these characteristics with survival and emphasizing the need for tailored post‐transplant management strategies to optimize outcomes.

## 1. Introduction

Liver transplantation (LT) is a life‐saving therapy for patients​ with severe end‐stage liver disease, significantly extending life expectancy and improving quality of life [1]. The limited availability of deceased donor livers and the growing number of patients on the transplant waitlist have led to increased waitlist mortality [2]. Globally, an estimated tens of thousands of liver transplants are carried out each year, with the United States, China, and Brazil doing more than any other country [3]. Although more than 10,000 liver transplants were carried out in the United States in 2023, this is a small portion of the demand worldwide [4]. While viral hepatitis continues to be a major cause in Asia, South America, and Africa, nonalcoholic fatty liver disease and alcohol‐associated liver disease are the most common reasons for transplantation in North America and Europe [3, 5]. In response, efforts have focused on expanding the donor pool and refining liver allocation policies to reduce waitlist deaths and ensure optimal post‐transplant outcomes [6, 7].

Survival outcomes after LT are influenced by a variety of clinical and demographic factors, including recipient age, donor age, sex, comorbidities (e.g., diabetes and cardiovascular disease [CVD]), model for end‐stage liver disease (MELD) score, donor organ quality, cold ischemia time, perioperative clinical status, and a prior transplantation [7, 8]. The MELD score has long served as the basis for liver allocation, prioritizing patients by the severity of liver dysfunction [9, 10]. Despite its utility in pretransplantation prioritization, MELD performs poorly in predicting post‐transplant survival [11]. To address this, MELD has undergone several updates, most recently MELD 3.0, which aims to improve prediction and address disparities, including those related to sex [6].

With a growing aging population, the proportion of older adults undergoing LT has increased. Both the American Association for the Study of Liver Diseases (AASLD) and the European Association for the Study of the Liver (EASL) emphasize the importance of considering physiological age over chronological age when considering transplant candidacy [8, 12]. Evidence suggests that carefully selected older recipients can achieve comparable perioperative and long‐term outcomes to younger adults [13, 14]. Moreover, chronic kidney disease (CKD) and diabetes mellitus (DM), particularly when dialysis is necessary, are also independently related to increased post‐transplant mortality and graft failure; dialysis dependence carries the highest risk at any age [15–17]. Nonetheless, comprehensive pretransplant assessment, including the evaluation of frailty, sarcopenia, and metabolic comorbidities, remains essential for optimizing outcomes [12].

We aim to evaluate the impact of various clinical factors on the survival benefit among liver transplant recipients. Specifically, we examined the years of life gained following LT, stratified by age at transplant and other key clinical variables. By identifying predictors of survival benefit of LT, this study aims to enhance the understanding of LT outcomes and support informed decision‐making in the context of organ scarcity.

## 2. Materials and Methods

### 2.1. Data Source

This study used data from the Scientific Registry of Transplant Recipients (SRTR). The SRTR data system includes data on all donor, wait‐listed candidates, and transplant recipients in the United States, submitted by the members of the organ procurement and transplantation network (OPTN) [18]. The Health Resources and Services Administration (HRSA), U.S. Department of Health and Human Services, provides oversight to the activities of the OPTN and SRTR contractors.

### 2.2. Ethical Statement

The study was ethically approved by the Research Ethics Board of University Health Network, under Approval Number REB: 21‐5339.

### 2.3. Study Population

We identified 101,770 individuals, including 82,514 transplanted and 19,256 nontransplanted patients. We included patients 18 years of age and older with a transplantation date between January 1, 2003, and February 29, 2020, and nontransplanted patients listed between January 2, 2003, and February 27, 2020.

Patients were followed to the earliest of death, loss to follow‐up, or the end of the observation period in March 2021. Post‐transplant follow‐up was a minimum of 1 year.

Patients who were listed for transplantation outside of the study period, survived less than a year after transplantation, or dropped out during that time were excluded from the analysis. Additionally, patients with a follow‐up period of zero and pediatric patients were excluded from the study.

In the transplanted group, patients with a last follow‐up status of “re‐transplanted” or “not seen” were excluded. For the nontransplanted group, patients whose last follow‐up status was other than “alive” or “death” were also excluded (Table S1, S2).

### 2.4. Patient Outcomes

Life expectancy and post‐transplant survival were the primary outcomes. By comparing the life expectancy of liver transplant recipients with that of matched nontransplanted individuals, we assessed the survival benefit. Estimates of life expectancy were categorized by the MELD score and age at transplant. Long‐term post‐transplant survival and the effect of recipient characteristics on mortality were secondary outcomes. Using multivariable survival models, the relationship between demographic and clinical factors, including age, sex, body mass index (BMI), comorbidities (e.g., diabetes, hypertension, and CVD), and MELD components, and survival, was evaluated.

#### 2.4.1. Consistency of MELD‐Na Scores in the Study Cohort

Since the original MELD was released in 2002, MELD‐Na was adopted into U.S. allocation in 2016, and MELD 3.0 was put into effect in 2023 [6, 19–21]. We retrospectively transformed the original MELD scores into MELD‐Na scores for data from 2002 to 2020 as the SRTR database did not include MELD‐Na values. This conversion was used consistently over the whole study period, including after 2016. To calculate the MELD‐Na, the following formula was utilized:

If MELD > 11, then otherwise, MELD‐Na was set equal to the original MELD score. Additionally, MELD‐Na values greater than 40 were capped at 40 to maintain clinical relevance.

(1) MELD−Na=MELD+1.32×137−Na−0.033×MELD×137−Na.

#### 2.4.2. Liver Disease Etiology and Patient Liver Status

The underlying causes of liver disease were divided into the following categories: acute hepatic necrosis (AHN), which includes viral hepatitis and other causes like fulminant autoimmune hepatitis or acute viral infection; alcohol‐related liver disease, which includes alcoholic cirrhosis, alcoholic cirrhosis with Hepatitis C, and acute alcoholic hepatitis; biliary diseases, which include primary biliary cholangitis (PBC), primary sclerosing cholangitis (PSC), and other biliary disorders; cirrhosis brought on by chronic liver diseases, such as viral hepatitis, autoimmune liver diseases, metabolic dysfunction‐associated steatohepatitis (MASH), drug‐induced liver injury, cryptogenic causes, and other specific conditions such as histiocytosis, sarcoidosis, and granulomatosis; and lastly, an additional category that includes all liver diseases not included in the previously mentioned groups.

### 2.5. Analytical Approach

Descriptive analyses were performed where counts and proportions were provided for categorical variables, whereas mean and standard deviation (SD) was estimated for the continuous variables for the whole cohort as well as deceased versus alive patients. The difference between the two groups was assessed using chi‐squared tests or Fisher’s exact tests for categorical variables and two‐sample *t* tests or Wilcoxon tests were used for continuous variables, depending on the data distribution.

Multivariable survival analyses were performed using the Cox proportional hazards model and Weibull models. The Cox proportional hazards model and the Weibull model were fit to the data using time‐to‐event as the outcome variable and the pretransplant factors as predictor variables. The hazard ratio (HR) represents the relative risk of the event (death) associated with a one‐unit change in the predictor variable. The Cox model was used to examine the risk factors associated with post‐transplant mortality, and the results were presented in the form of HR with its 95% confidence interval (CI).

In order to examine survival benefits of liver transplant, we used the propensity score matching (PSM) method to perform one‐to‐one matching between transplanted and nontransplanted groups based on gender, diagnosis (MASH vs. others), diabetes status, age, MELD‐Na, and BMI. The standardized mean difference was assessed after matching.

Life expectancy was analyzed by calculating restricted mean survival time (RMST), which is defined as the area under the Kaplan–Meier survival curve up to 16 years post‐transplant. It can be interpreted as the average survival time or life expectancy during a defined time period [22]. Prior to calculating the RMST, we performed PSM to balance the characteristics between the transplanted and nontransplanted groups.

The Weibull model was used to determine the variables significantly associated with post‐transplant survival time, and the results were provided in the form of event time ratio (ETR) with its 95% CI. A *p* value of < 0.05 was considered statistically significant.

All analyses were conducted using SAS, Version 9.4 (SAS Institute, Cary, NC). A *p* value of < 0.05 was considered statistically significant.

## 3. Results

### 3.1. Descriptive Statistics

The final study population comprised 101,770 individuals, including 82,514 transplanted and 19,256 nontransplanted patients.

Descriptive statistics for the transplanted study participants are shown in Table 1. Participants were transplanted from 2003 to 2020, with a median follow‐up time of 1674 days (range 1–5840 days). Females made up 33.67% of the study population. The average age of the cohort was 54.46 ± 10.59 years, with close to 90% of the participants being over the age of 40 years and over 55% being between the ages of 40 and 60 years.

**Table 1:** Outcomes and characteristics of liver transplant recipients by survival status.

Variable	Category/statistic	Alive (*N* = 62,258)	Deceased (*N* = 20,256)	Total (*N* = 82,514)	*p* value
Transplant time period	2003–2005	4252 (06.83%)	3740 (18.46%)	7992 (9.69%)	< 0.001
	2006–2010	13,289 (21.35%)	8934 (44.11%)	22,223 (26.93%)	
	2011–2015	18,335 (29.45%)	5400 (26.66%)	23,735 (28.76%)	
	2016–2020	26,382 (42.38%)	2182 (10.77%)	28,564 (34.62%)	

Age at transplant (years)	Mean (SD)	54.16 (10.76)	55.37 (9.98)	54.46 (10.59)	< 0.001
	*n* (*n* Missing)	62,258 (0)	20,256 (0)	82,514 (0)	
	18 to 29	2398 (3.85%)	555 (2.74%)	2953 (3.58%)	< 0.001
	30 to 49	14,218 (22.84%)	3887 (19.19%)	18,105 (21.94%)	
	50 to 69	43,791 (70.34%)	14,961 (73.86%)	58,752 (71.20%)	
	≥ 70	1851 (2.97%)	853 (4.21%)	2704 (3.28%)	
	Missing	0 (0%)	0 (0%)	0 (0%)	

Patient’s sex	Female	21,644 (34.77%)	6142 (30.32%)	27,786 (33.67%)	< 0.001
	Male	40,614 (65.23%)	14,114 (69.68%)	54,728 (66.33%)	

Patient’s education status	Highschool or less	27,108 (47.89%)	9079 (53.84%)	36,187 (49.26%)	< 0.001
	None	199 (00.35%)	58 (0.34%)	257 (0.35%)	
	Postsecondary	24,946 (44.07%)	6631 (39.32%)	31,577 (42.98%)	
	Postgraduate	4347 (07.68%)	1096 (6.50%)	5443 (7.41%)	
	Missing	5658 (9.09%)	3392 (16.75%)	9050 (10.97%)	

Height at transplant (cm)	Mean (SD)	171.84 (10.29)	172.63 (10.24)	172.03 (10.28)	< 0.001
	*n* (*n* Missing)	62,193 (65)	20,232 (24)	82,425 (89)	

Recipient weight at transplant (kg)	Mean (SD)	84.22 (19.80)	84.24 (19.55)	84.22 (19.74)	0.93
	*n* (*n* Missing)	61,583 (675)	19,801 (455)	81,384 (1130)	

BMI at transplant	Mean (SD)	28.43 (5.84)	28.19 (5.79)	28.37 (5.83)	< 0.001
	*N* (*N* Missing)	61,541 (717)	19,784 (472)	81,325 (1189)	

Categorical BMI at transplant	Normal	17,448 (28.35%)	5769 (29.16%)	23,217 (28.55%)	< 0.001
	Obesity	21,691 (35.25%)	6594 (33.33%)	28,285 (34.78%)	
	Overweight	21,246 (34.52%)	6992 (35.34%)	28,238 (34.72%)	
	Underweight	1156 (1.88%)	429 (2.17%)	1585 (1.95%)	
	Missing	717 (01.15%)	472 (2.33%)	1189 (1.44%)	

Clinical measures					
Diagnosis					
Etiology of liver disease	Acute hepatic necrosis (AHN) (viral hepatitis, other, specify (e.g., acute viral infection, autoimmune hepatitis‐fulminant	2593 (4.16%)	685 (3.38%)	3278 (3.97%)	< 0.001
	Alcohol‐related liver disease (alcoholic cirrhosis, alcoholic cirrhosis with Hepatitis C, and acute alcoholic hepatitis)	13,305 (21.37%)	4062 (20.05%)	17,367 (21.05%)	
	Biliary disease (PBC, PSC, other)	05,799 (9.31%)	1358 (6.70%)	7157 (8.67%)	
	Cirrhosis results from chronic liver diseases (viral hepatitis, autoimmune, MASH, drug induced, cryptogenic, other specify (e.g., histiocytosis, sarcoidosis, granulomatosis)	33,703 (54.13%)	11,640 (57.46%)	45,343 (54.95%)	
	Other	6858 (11.02%)	2511 (12.40%)	9369 (11.35%)	
Patient diabetes status	Yes	15,255 (24.71%)	6078 (30.50%)	21,333 (26.12%)	< 0.001
Peptic ulcer disease	Yes	1311 (04.11%)	731 (04.71%)	2042 (04.31%)	0.002
Coronary artery disease	Yes	1311 (4.11%)	731 (4.71%)	2042 (4.31%)	0.002
Drug treated systemic hypertension	Yes	8037 (24.64%)	4416 (28.11%)	12,453 (25.77%)	< 0.001
Symptomatic cerebrovascular disease	Yes	264 (0.80%)	161 (1.02%)	425 (0.87%)	0.016^∗^
Symptomatic peripheral vascular disease	Yes	317 (0.97%)	237 (1.50%)	554 (1.14%)	< 0.001
Spontaneous bacterial peritonitis	Yes	6466 (10.45%)	2103 (10.49%)	8569 (10.46%)	0.86
History of portal vein thrombosis	Yes	3377 (5.55%)	832 (4.31%)	4209 (5.25%)	< 0.001
Last measurement on waitlist					
Candidate last serum creatinine (mg/dL)	Mean (SD)	1.54 (1.36)	1.61 (1.39)	1.56 (1.37)	< 0.001
	*n* (*n* Missing)	62,241 (17)	20,246 (10)	82,487 (27)	
Candidate last international normalized ratio (INR)	Mean (SD)	1.88 (0.91)	1.76 (0.81)	1.85 (0.89)	< 0.001
	*n* (*n* Missing)	62,093 (165)	20,211 (45)	82,304 (210)	
Candidate last albumin (g/dL)	Mean (SD)	3.08 (0.72)	2.99 (0.71)	3.06 (0.72)	< 0.001
	*n* (*n* Missing)	62,122 (136)	20,206 (50)	82,328 (186)	
Candidate last bilirubin (μmol/L)	Mean (SD)	8.65 (11.08)	7.48 (10.30)	8.36 (10.91)	< 0.001
	*n* (*n* Missing)	62,234 (24)	20,245 (11)	82,479 (35)	
Candidate last serum sodium (mmol/L)	Mean (SD)	136.05 (5.19)	136.25 (5.08)	136.10 (5.17)	< 0.001
	*N* (*N* Missing)	58,264 (3994)	16,758 (3498)	75,022 (7492)	
Candidate last SRTR MELD given	Mean (SD)	22.63 (9.99)	21.38 (9.80)	22.34 (9.96)	< 0.001
	*n* (*n* Missing)	58,873 (3385)	17,332 (2924)	76,205 (6309)	
Categorical last SRTR MELD given	< 19	23,446 (39.82%)	7710 (44.48%)	31,156 (40.88%)	< 0.001
	≥ 19	35,427 (60.18%)	9622 (55.52%)	45,049 (59.12%)	
	Missing	3385 (5.44%)	2924 (14.44%)	6309 (7.65%)	
Dialysis within week of transplant	No	54,239 (87.25%)	17,913 (88.76%)	72,152 (87.62%)	< 0.001
	Yes	7927 (12.75%)	2268 (11.24%)	10,195 (12.38%)	
	Missing	92 (00.15%)	75 (0.37%)	167 (0.20%)	
Pretransplant: ALT (U/L)	Mean (SD)	145.07 (577.50)	128.90 (462.84)	139.49 (540.77)	0.001
	*N* (*N* Missing)	29,828 (32,430)	15,696 (4560)	45,524 (36,990)	
Categorical pretransplant ALT	< 40	12,494 (41.89%)	6738 (42.93%)	19,232 (42.25%)	0.003
	≥ 1000	712 (2.39%)	309 (1.97%)	1021 (2.24%)	
	≥ 40 and < 1000	16,622 (55.73%)	8649 (55.10%)	25,271 (55.51%)	
	Missing	32,430 (52.09%)	4560 (22.51%)	36,990 (44.83%)	
Post‐transplant					
Time to last follow‐up (days) (death, alive, lost to follow‐up)	Mean (SD)	2109.67 (1598.58)	1686.43 (1380.36)	2005.77 (1558.54)	< 0.001
	*N* (*N* Missing)	62,258 (0)	20,256 (0)	82,514 (0)	

*Note:* Data stratified by time, demographics, comorbidities, clinical variables, last measurement on waitlist.

Abbreviations: MASH, metabolic dysfunction–associated steatohepatitis; PBC, primary biliary cholangitis; PSC, primary sclerosing cholangitis; SD, Standard deviation; SRTR, Scientific Registry of Transplant Recipients.

^∗^Indicates statistical significance at *p* <0.05.

Over 49% of LT recipients had high school or less as their highest level of education. The average BMI at transplant was 28.37 ± 5.83 with over 70% of individuals being overweight or obese. According to the histological outcome, cirrhosis was the primary reason why most of the patients in this research needed liver transplants. The most common diagnosis was cirrhosis (54.95%), with a smaller proportion of the cohort having MASH (9.81%). Just over 26% of the individuals had diabetes. The average model for the MELD‐Na score at transplant was 22.34 ± 9.96. The number of patients who were lost to follow‐up among those who underwent transplantation is 4445 (5.39%) (Table S3). Meanwhile, the number of transplanted patients with five or more years of follow‐up is 37,772 (45.78%); in the nontransplanted group, this number is 2011 (10.44%) (Table S4, S5).

A total of 534 patients died from cardiovascular events: 268 (50.9%) in the nontransplanted group and 266 (49.81%) in the transplanted group. It means that 6.98% of transplanted patients and 7.75% of nontransplanted patients died from CV causes. Furthermore, cardiovascular death causes comprised 3.66% of the total death rate in the transplanted group and 3.69% of the total death rate in the nontransplant group (Table S6).

Table 2 summarizes the descriptive statistics for both patient populations (transplanted and nontransplanted) after PSM. The average age after matching was 55.59 years, with a male predominance of 62.79%. Mean baseline MELD after matching was 13.67, while mean baseline BMI was 28.82. After matching, the standardized mean differences for all covariates were below 0.1, indicating excellent balance and negligible differences in covariate distributions between the transplanted and nontransplanted groups. Thus, the matched groups are highly comparable, allowing for a valid analysis of transplant versus nontransplant survival outcomes.

**Table 2:** Descriptive statistics for transplanted and nontransplanted patient populations after matching.

Variable	Category/statistic	Not transplanted (*N* = 18,898)	Transplanted (*N* = 18,898)	Total (*N* = 37,796)	Standardized mean difference
Age at transplant (years)	Mean (SD)	55.34 (9.44)	55.84 (10.45)	55.59 (9.96)	0.05
	*N* (*N* Missing)	18,898 (0)	18,898 (0)	37,796 (0)	

Baseline MELD	Mean (SD)	13.41 (4.56)	13.92 (5.29)	13.67 (4.95)	0.1
	*N* (*N* Missing)	18,898 (0)	18,898 (0)	37,796 (0)	

Baseline BMI	Mean (SD)	28.98 (5.88)	28.66 (5.72)	28.82 (5.80)	0.06
	*N* (*N* Missing)	18,898 (0)	18,898 (0)	37,796 (0)	

Patient’s sex	Female	7446 (39.40%)	6618 (35.02%)	14,064 (37.21%)	0.06
	Male	11,452 (60.60%)	12,280 (64.98%)	23,732 (62.79%)	
	Missing	0 (0%)	0 (0%)	0 (0%)	

Primary diagnosis	MASH	2531 (13.39%)	2189 (11.58%)	4720 (12.49%)	0.06
	Other	16,367 (86.61%)	16,709 (88.42%)	33,076 (87.51%)	
	Missing	0 (0%)	0 (0%)	0 (0%)	

Patient’s education status	Highschool or less	8651 (52.11%)	8498 (49.22%)	17,149 (50.63%)	0.06
	Postsecondary or Postgraduate	7952 (47.89%)	8768 (50.78%)	16,720 (49.37%)	
	Missing	2295 (12.14%)	1632 (8.64%)	3927 (10.39%)	

Patient diabetes status	No	12,984 (68.71%)	13,272 (70.23%)	26,256 (69.47%)	0.03
	Yes	5914 (31.29%)	5626 (29.77%)	11,540 (30.53%)	
	Missing	0 (0%)	0 (0%)	0 (0%)	

Coronary artery disease	No	9353 (96.90%)	10,213 (96.93%)	19,566 (96.91%)	0
	Yes	299 (3.10%)	324 (3.07%)	623 (3.09%)	
	Missing	9246 (48.93%)	8361 (44.24%)	17,607 (46.58%)	

Drug treated systemic hypertension	No	7129 (71.33%)	7773 (71.73%)	14,902 (71.54%)	0
	Yes	2865 (28.67%)	3063 (28.27%)	5928 (28.46%)	
	Missing	8904 (47.12%)	8062 (42.66%)	16,966 (44.89%)	

*Note:* MELD, model for end‐stage liver disease.

Abbreviations: BMI, body mass index; MASH, metabolic dysfunction–associated steatohepatitis; SD, standard deviation.

### 3.2. Survival Benefit of Transplant Stratified by Age of Transplant

Survival benefit from transplant by comparing survival of the transplanted cohort versus nontransplanted cohort stratified by age is illustrated in Figure 1 and Table 3. Transplanted patients in the 18–29‐year‐old age group exhibited a life expectancy of 12.57 years, while their nontransplanted counterparts had a life expectancy of 7.64 years, yielding a survival benefit of 4.93 years (*p* < 0.0001). This statistically significant trend was consistent across all age groups with patients transplanted in the 30–49‐year‐old age group exhibiting a survival benefit of 6.43 years relative to their nontransplanted counterparts (12.24 years vs. 5.81 years, *p* < 0.0001). Patients transplanted in the 50–69‐year‐old age group exhibited a survival benefit of 6.30 years relative to their nontransplanted counterparts (11.11 years vs. 4.81 years, *p* < 0.0001). Patients transplanted in their 70s and beyond exhibited a survival benefit of 3.67 years relative to their nontransplanted counterparts (6.49 years vs. 2.82 years, *p* < 0.0001).

Figure 1Liver transplant survival benefit stratified by age at transplant. (a) 18–29 years, (b) 30–49 years, (c) 50–69 years, (d) 70 years and above.

**Figure:**
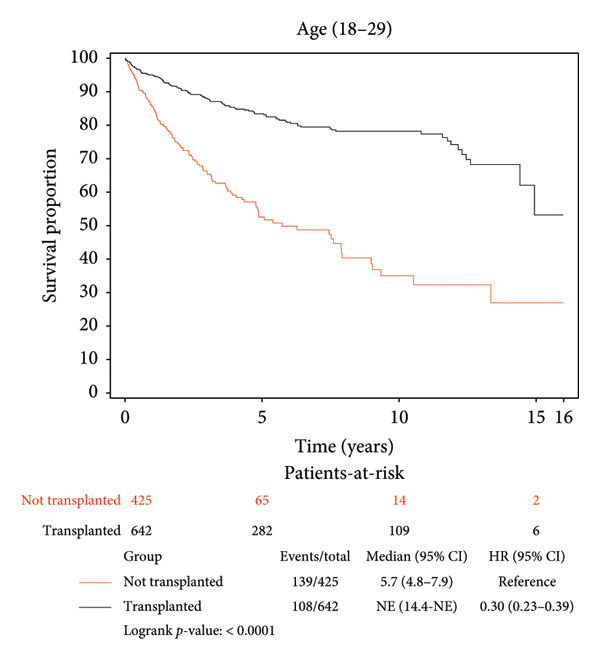
(a)

**Figure:**
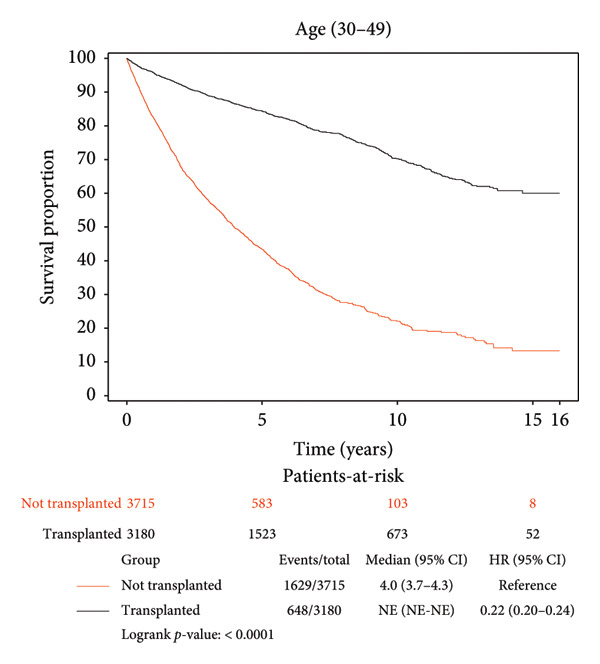
(b)

**Figure:**
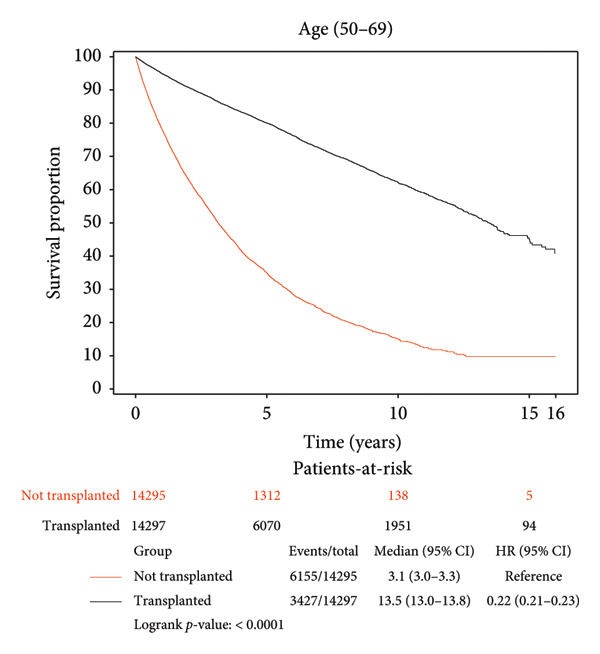
(c)

**Figure:**
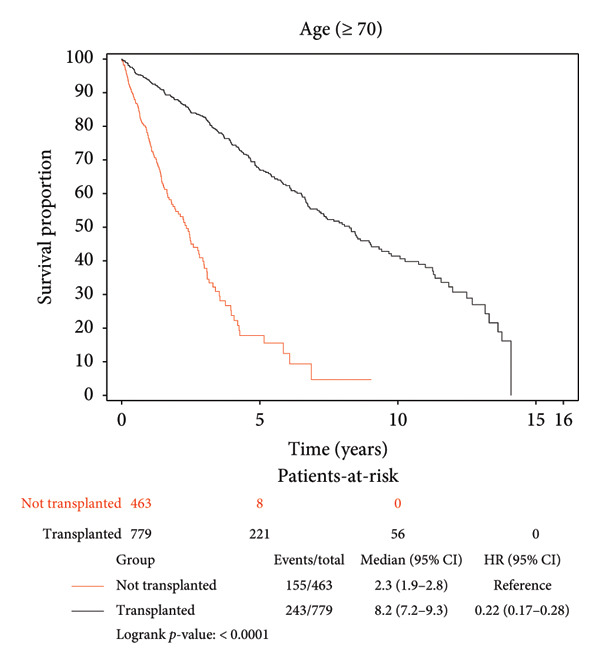
(d)

**Table 3:** Life expectancy via restricted mean survival time (RMST) and survival benefit of liver transplant stratified by age and model for end‐stage liver disease (MELD) score.

Variable	Life expectancy (years)		*p* value	Survival benefit (years)
	Nontransplanted	Transplanted		
Age group				
18–29	7.64	12.57	< 0.0001	4.93
30–49	5.81	12.24	< 0.0001	6.43
50–69	4.81	11.11	< 0.0001	6.30
≥ 70	2.82	6.49	< 0.0001	3.67
MELD score				
6–14	5.42	11.12	< 0.0001	5.7
15–19	4.45	11.07	< 0.0001	6.62
20–29	3.87	9.26	< 0.0001	5.4
30–40	3.86	9.71	< 0.0001	5.85

### 3.3. Survival Benefit of Transplant Stratified by MELD Score

Survival benefit from transplant by comparing survival of the transplanted cohort versus nontransplanted cohort stratified by the MELD score is illustrated in Figure 2 and Table 3. Transplanted patients with MELD scores of 6–14 exhibited a life expectancy of 11.12 years, while their nontransplanted counterparts had a life expectancy of 5.42 years, yielding a survival benefit of 5.7 years (*p* < 0.0001). This statistically significant trend was consistent across all MELD scores, with patients transplanted with MELD scores of (15–19) exhibiting the maximum survival benefit of 6.62 years relative to their nontransplanted counterparts (11.07 years vs. 4.45 years, *p* < 0.0001). Patients transplanted with MELD scores of (20–29) exhibited a survival benefit of 5.39 years relative to their nontransplanted counterparts (9.26 years vs. 3.87 years, *p* < 0.0001). Patients transplanted with MELD scores of (30–40) exhibited a survival benefit of 5.85 years relative to their nontransplanted counterparts (9.71 years vs. 3.86 years, *p* < 0.0001).

Figure 2Liver transplant survival benefit stratified by model for end‐stage liver disease (MELD) score at transplant. (a) MELD (6–14), (b) MELD (15–19), (c) MELD (20–29), (d) MELD (30–40).

**Figure:**
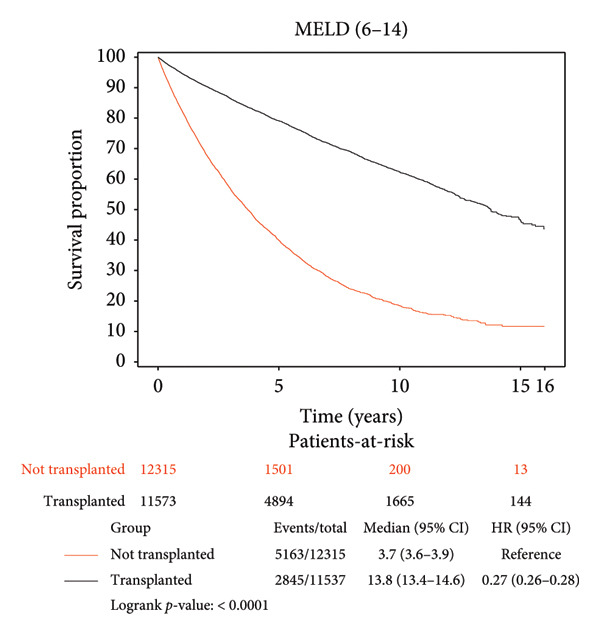
(a)

**Figure:**
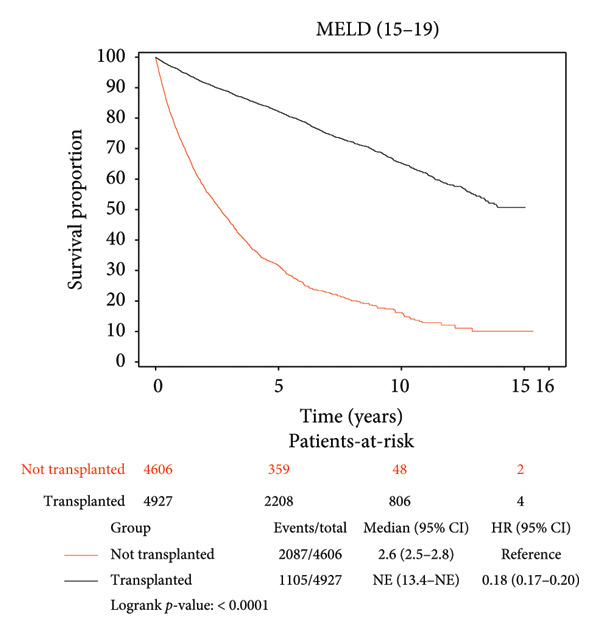
(b)

**Figure:**
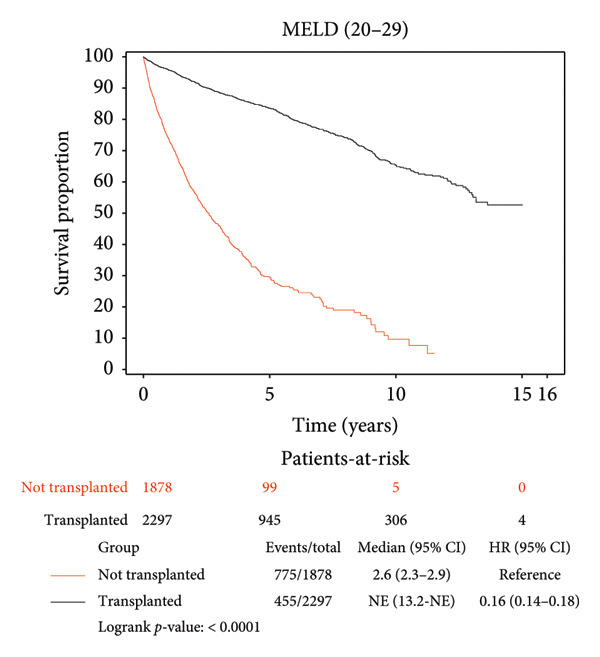
(c)

**Figure:**
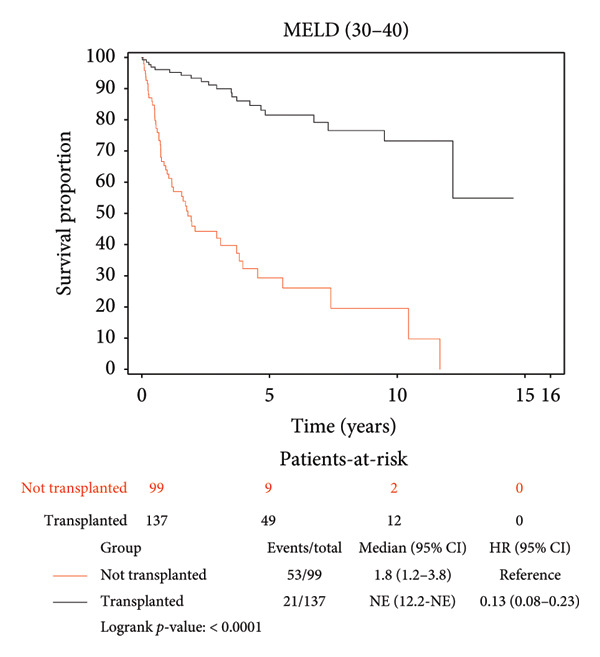
(d)

### 3.4. Long‐Term Outcomes Postliver Transplant for Recipients

**Figure 3:**
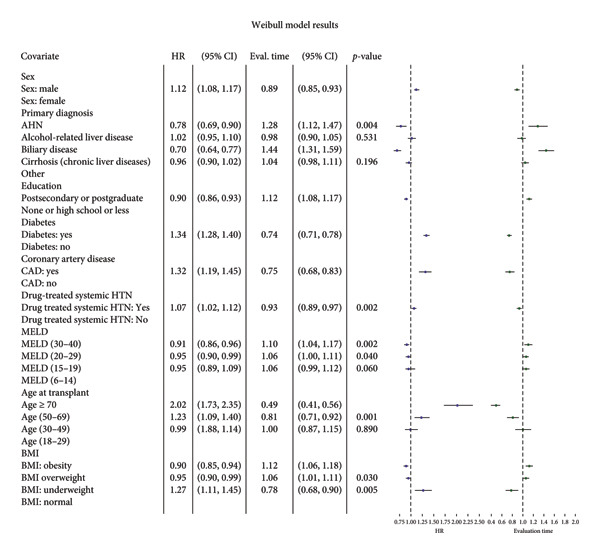
Multivariable survival analysis—Weibull model.

**Figure 4:**
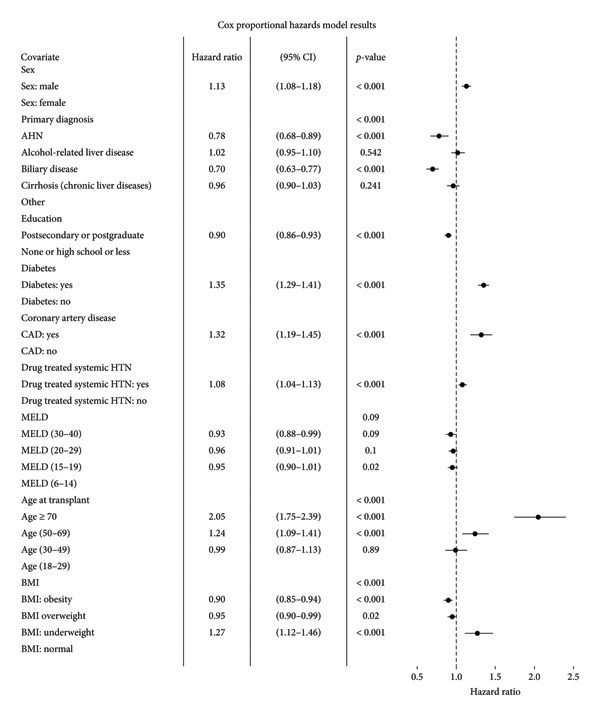
Multivariable survival analysis—Cox proportional model.

The multivariable Weibull model (Figure 3) showed that male patients had a higher instantaneous risk of mortality relative to female patients (HR 1.13, 95% CI 1.08–1.18, *p* < 0.001). Recipients of liver transplants aged 60 years and above showed 1.32 times increased instantaneous risk of mortality compared to the 18–40‐year‐old age cohort (HR 1.32, 95% CI 1.21–1.45, *p* < 0.001). Patients with diabetes and hypertension exhibited an increased risk of death (HR 1.31, 95% CI 1.25–1.37, *p* < 0.001 and HR 1.06, 95% CI 1.01–1.11, *p* = 0.02, respectively). The presence of coronary artery disease (CAD) was also linked to a 32% increased risk of mortality (HR 1.32, 95% CI 1.20–1.46, *p* < 0.001). Education had a protective effect; patients with postsecondary education had improved survival rates of 11% over those with lower education levels (HR 0.89, 95% CI 0.86–0.93, *p* < 0.001). Obesity was surprisingly associated with an 8% lower mortality risk (HR 0.92, 95% CI 0.87–0.97, *p* = 0.001), while being underweight was linked to increased mortality (HR 1.29, 95% CI 1.12–1.47, *p* < 0.001). Within the MELD components, higher last serum creatinine levels were associated with an increased mortality risk (HR 1.04, 95% CI 1.02–1.05, *p* < 0.001). The Cox proportional hazard model was used to validate the results by the Weibull model, and the results were concordant (Figures 3 and 4).

The event time ratio (ETR) sheds light on the differential impact of factors on survival time post‐transplant (Figure 3). Males showed a disadvantage (ETR 0.88, 95% CI 0.84–0.92, *p* < 0.001), indicating a 12% shorter survival time compared to females. Patients aged 60 years and above had a 25% shorter survival time compared to younger cohorts (ETR 0.75, 95% CI 0.68–0.82, *p* < 0.001). Compared to a grouping of all the other conditions, primary diagnoses of AHN and biliary disease were associated with significantly longer survival times (ETR 1.23, 95% CI 1.07–1.42, *p* = 0.004 and ETR 1.43, 95% CI 1.29–1.58, *p* < 0.001, respectively). Higher education levels were linked to improved survival time (ETR 1.12, 95% CI 1.08–1.17, *p* < 0.001). Diabetes is associated with 24% shorter survival time (ETR 0.76, 95% CI 0.72–0.79, *p* < 0.001). CAD and hypertension were indicative of reduced survival times (ETR 0.75, 95% CI 0.68–0.83, *p* < 0.001) and (ETR 0.94, 95% CI 0.90–0.99, *p* = 0.02), respectively. Regarding the BMI, obesity was again surprisingly associated with a 9% improvement in survival time over those with a normal BMI (ETR 1.09, 95% CI 1.04–1.15, *p* = 0.001). On the other hand, being underweight was linked to a decreased survival time of 22% (ETR 0.77, 95% CI 0.67–0.89, *p* = 0.0003). For MELD components, a higher international normalized ratio (INR) signified a longer survival time, while a higher serum creatinine signified a shorter survival time (ETR 1.09, 95% CI 1.06–1.13, *p* < 0.001) and (ETR 0.96, 95% CI 0.95–0.97, *p* < 0.001), respectively.

## 4. Discussion

In this nationwide retrospective cohort study, we evaluated the impact of demographic and clinical factors on survival in a cohort of 101,770 patients from the SRTR, with up to 16 years of follow‐up. We found that several factors at the time of transplantation significantly influenced survival, including age, sex, BMI, MELD scores, education level, underlying liver disease, and comorbidities (such as diabetes and hypertension). Our findings underscore LT’s profound survival benefit while highlighting significant predictors of post‐transplant outcomes and their implications for clinical practice.

A strong and statistically significant association was observed between age at the time of LT and post‐transplant survival. Our study demonstrated that LT offers significant survival benefits across all age groups, although the magnitude of this benefit diminishes with increasing recipient age. For instance, patients transplanted in their 20s had a post‐LT life expectancy of 12.57 years, compared to 6.49 years for those transplanted in their 70s. When comparing transplanted and nontransplanted individuals, the survival benefit was substantial across all age groups, with the greatest gain observed among recipients aged between 30 and 70 years. While patients aged 70 years and above still experienced a meaningful benefit of 3.67 years, this was the smallest among the groups, reflecting the nuanced impact of age on survival outcomes. These findings underscore the importance of individualized, age‐informed risk‐benefit analysis in transplant decision‐making.

Our findings corroborate prior work by Pischke et al., who identified age as a negative predictor of post‐LT survival, and Chen et al., who reported a 17.92% risk of death in recipients over 60 years compared to 12.77% in those under 60 years, 5 years post‐LT [17, 23]. These findings collectively highlight the challenges of transplanting older patients, where factors such as frailty, comorbidities, and reduced physiological reserves likely contribute to diminished outcomes. Nevertheless, our results emphasize that while younger recipients naturally have longer post‐transplant life expectancy, middle‐aged adults may experience the greatest relative survival gain compared to their nontransplant counterparts. Even among the oldest cohort (70 years and above), LT significantly extends life expectancy, reaffirming its life‐saving values across the age spectrum and supporting its use in carefully selected older populations.

Identifying sex differences in survival outcomes across age groups may offer valuable insights into the management of LT [24]. Our findings revealed notable sex‐based variations in long‐term post‐transplant outcomes. Males were found to have a 1.13 higher chance of instantaneous risk of mortality and a 12% reduced survival time than females. There are various studies reporting better survival benefits in women. In a large nationwide analysis of sex differences in Spain, female LT recipients had an overall lower probability of death compared to males [25]. Female sex was associated with better survival in patients aged over 65 years undergoing LT, but not in older men [26]. Better women outcomes were considerable and statistically significant for the 50–65‐year age group of recipients of deceased donor LT [27]. However, recent studies reported similar post‐transplant overall survival in both males and females [28, 29]. Therefore, the observation of sex‐specific survival differences warrants further investigation to elucidate underlying biological, clinical, or social determinants contributing to this result.

The presence of comorbidities, including diabetes, hypertension, and CVD, was associated with reduced post‐transplant survival and increased mortality risk in our study. These findings are consistent with previous research on long‐term outcomes in patients with post‐transplant diabetes [30, 31]. They also align with the work of Rinella et al., who demonstrated that pretransplant diabetes has a significant negative prognostic impact on post‐LT survival in patients with MASH cirrhosis [32].

Surprisingly, obesity was associated with a lower mortality risk and improved survival time in our study, contradicting previous studies, which showed decreased survival with increasing BMI in LT recipients [33, 34]. Conversely, underweight status was linked to increased mortality and shorter survival times. This observation is amplified by research on underweight Asian patients, where Lee et al. found significantly poor overall survival compared to non‐underweight individuals [35]. Furthermore, a meta‐analysis on the BMI and liver transplant outcomes reported that underweight recipients face an increased risk of post‐transplant death and graft loss [33]. These findings underscore the potential benefits of targeted interventions such as nutritional support, physical exercise, prehabilitation, and early and continuous rehabilitation post‐transplant for patients at both extreme of the BMI spectrum.

Moreover, a cohort study of 2007 living donor liver transplant recipients from a single center in Korea showed no significant association between post‐transplant survival and education level [36]. In contrast, a more recent study reported a direct relationship between educational attainment and survival among liver transplant recipients with MASH [30]. The protective effect of higher education observed in our study is consistent with broader public health research, which underscores the critical role of social determinants, such as education, in shaping health outcomes. In summary, substantial survival benefits were found across all age groups although the magnitude of this benefit decreases with advancing recipient age. This study reinforces the transformative impact of LT on survival outcomes and highlights the nuanced interplay of patients’ demographic, clinical, and social factors in shaping post‐transplant trajectories. By addressing the identified predictors, the transplant can further enhance the life‐saving potential of LT.

Moreover, the findings enable hepatologists and transplant teams’ reliable evidence to back up the selection of fair and rational liver transplant recipients through interdisciplinary decision‐making procedures. In partnership with the identification of important prognostic factors such as demographics, comorbidities, and disease etiology, the demonstrated survival benefits across all age groups and MELD scores allow transplant committees to make transparent, evidence‐based allocation decisions that go beyond subjective evaluations [37]. In order to maximize both individual outcomes and organ utilization efficiency, hepatologists can methodically weigh various patient factors by integrating this thorough survival data into multidisciplinary reviews. This evidence‐based strategy guarantees fair access to transplantation while upholding strict selection standards that optimize the survival benefit of each organ allotted.

### 4.1. Strengths and Limitations

This study’s strengths include its large sample size and comprehensive data spanning multiple decades, enhancing the generalizability of our findings. The use of advanced statistical techniques, including Cox proportional hazard models and Weibull models, provided robust insights into factors influencing post‐LT survival. Additionally, the application of RMST allowed for a detailed quantification of life expectancy gains, offering a clinically meaningful metric for assessing transplant benefits.

However, the study has limitations. As a retrospective cohort study, it is inherently subject to biases related to unmeasured confounding. While PSM minimized these biases, causality cannot be definitively established. Additionally, the dataset’s observational window was limited to 16 years, potentially underestimating long‐term survival benefits. The lack of detailed donor data, such as quality and match characteristics, further limits the ability to assess their impact on recipient outcomes.

Moreover, we acknowledge that classifying pivotal liver disease etiologies into wide‐ranging categories may mask important heterogeneity between patients, such as chronic viral hepatitis versus MASH, although both conditions develop into cirrhosis. While we employed the SRTR data dictionary to categorize conditions, the limitations within a large national database persist. These limitations can influence outcomes and lead to subtle differences between subgroups.

## 5. Conclusion

This study underscores the life‐saving impact of LT and the influence of demographic, clinical, and social factors on outcomes. By addressing the identified predictors, the transplant can further enhance the life‐saving potential of LT. Future research should explore the mechanistic basis of observed disparities and investigate interventions to mitigate negative prognostic factors like diabetes and CVD. Longitudinal studies with extended follow‐up periods are needed to capture the full spectrum of long‐term outcomes post‐LT. Additionally, integrating donor‐specific variables and leveraging machine learning models could enhance predictive accuracy for survival benefits and guide personalized transplant decisions.

**Nomenclature**
AASLD: American Association for the Study of Liver Diseases
AHN: Acute hepatic necrosis
ALT: Alanine aminotransferase
BMI: Body mass index
CKD: Chronic kidney disease
CAD: Coronary artery disease
CVD: Cardiovascular disease
CI: Confidence interval
DM: Diabetes mellitus
ETR: Event time ratio
EASL: European Association for the Study of the Liver
HR: Hazard ratio
HRSA: Health Resources and Services Administration
INR: International normalized ratio
LT: Liver transplant
MASH: Metabolic dysfunction‐associated steatohepatitis
MELD: Model for end‐stage liver disease
OPTN: Organ procurement and transplantation network
PSM: Propensity score matching
RMST: Restricted mean survival time
SRTR: Scientific Registry of Transplant Recipients
SD: Standard deviation

## Disclosure

The interpretation and reporting of these data are the responsibility of the author(s) and in no way should be seen as an official policy of or interpretation by the SRTR or the US Government.

## Conflicts of Interest

The authors declare no conflicts of interest.

## Author Contributions

Mamatha Bhat led the conceptualization of the study and oversaw data curation alongside Naomi Khaing Than Hlaing. The investigation was conducted by Bishoy Lawendy and Mouaid Alim, with methodological contributions from Shiyi Chen and Mamatha Bhat. The initial draft of the manuscript was written by Bishoy Lawendy, Mouaid Alim, Saba Maleki, and Mamatha Bhat, while all authors contributed to reviewing and editing the final version. Mouaid Alim and Bishory Lawendy contributed equally to this work.

## Funding

This study was supported by the UHN Foundation (Mamatha Bhat) and the NSERC USRA Program (Mouaid Alim).

## Supporting Information

Table S1: Number of patients excluded due to death within 1 year after transplant (transplanted patients); Table S2: number of patients excluded with “0” days follow‐up (listed and nontransplanted patients); Table S3: additional data such as number or proportion of patients lost to follow‐up; Table S4: number or proportion of patients with 5 or more years of follow‐up; Table S5: number or proportion of patients with 5 or more years of follow‐up; Table S6: causes of death by transplant.

## Supporting information


**Supporting Information** Additional supporting information can be found online in the Supporting Information section.

## Data Availability

The data reported here have been supplied by the Hennepin Healthcare Research Institute (HHRI) as the contractor for the Scientific Registry of Transplant Recipients (SRTR).
