# Supplementary material for: Impact of Diverse Clinical Characteristics on Survival Benefit of Liver Transplantation
Source: J Transplant. 2026 Jan 15;2026:1291289. doi: 10.1155/joot/1291289 (PMC12805940; doi:10.1155/joot/1291289)
Supplement: Supplementary file 1 — Supporting Information Additional supporting information can be found online in the Supporting Information section. [file JOOT-2026-1291289-s001.docx]

**Supplementary Material**

**Table S1. Number of patients excluded due to death within 1 year after transplant**

Transplanted patients

| **Exclusion criteria** | **N (excluded)** | **Remaining cohort** | **N (remaining)** |
| --- | --- | --- | --- |
|  |  | Patients transplanted in between October 1, 1987, to Mar 2, 2021 | 183721 |
| Remove patients listed outside time of interest | 57048 | Patients transplanted after Jan 1, 2003 | 126673 |
| Remove patients dead or dropout within 1 year | 30103 | Patients with at least 1 year of follow up | 96570 |
| Remove patient with last follow up status of “re-transplanted” or “not seen” | 6415 | Patients with last follow up status of Alive, Deceased or Lost to Follow up | 90155 |
| Remove pediatric patients | 7298 | Adult patients (age > 18-year-old) | 82857 |
| Remove patients with follow up time = 0 | 343 | Patients with valid survival time | 82514 |
|  |  | **Total included cohort** | 82514 |

**Table S2. Number of patients excluded with “0” days follow-up.**

Listed and non-transplanted patients

| **Exclusion criteria** | **N (excluded)** | **Remaining cohort** | **N (remaining)** |
| --- | --- | --- | --- |
|  |  | Patients listed in between October 1, 1987, to Mar 2, 2021 | 327212 |
| Remove patients listed outside time of interest | 107698 | Patients listed after Jan 1, 2003 | 219514 |
| Remove patients dead or dropout within 1 year | 161533 | Patients with at least 1 year of follow up | 57981 |
| Remove patient with last follow up status other than “death” or “alive” | 38316 | Patients with last follow up status of Alive and Deceased | 19665 |
| Remove pediatric patients | 353 | Adult patients (age > 18-year-old) | 19312 |
| Remove patients with follow up time = 0 | 56 | Patients with valid survival time | 19256 |
|  |  | **Total included cohort** | 19256 |

Transplanted patients combined with non-transplanted patients: 82514 + 19256 = 101770

**Table S3. Additional data such as number or proportion of patients lost to follow-up, % lost to follow up among transplanted patients (represented by L): n=4445, %=5.39%**

| **Last Status** | **Frequency** | **Percent** | **Cumulative Frequency** | **Cumulative Percent** |
| --- | --- | --- | --- | --- |
| **Alive** | **57773** | **70.2** | **57773** | **70.02** |
| **Death** | **202096** | **24.60** | **78069** | **94.61** |
| **Lost to follow-Up** | **4445** | **5.39** | **82514** | **100.00** |

% lost to follow up among non-transplanted patients:

Please see flow chart, no one lost to follow up. Either alive or death.

**Table S4. Number or proportion of patients with 5 or more years follow-up**

**Transplanted patients: follow up more than 5 years: n=37772, %=45.78%**

| **Follow up more than 5 years** | **Frequency** | **Percent** | **Cumulative Frequency** | **Cumulative Percent** |
| --- | --- | --- | --- | --- |
| **No** | **44742** | **54.22** | **44742** | **54.22** |
| **Yes** | **37772** | **45.78** | **82514** | **100.00** |

**Table S5. Number or proportion of patients with 5 or more years follow-up**

**Non-transplanted patients: follow up more than 5 years: n=2011, %=10.44%**

| **Follow up more than 5 years** | **Frequency** | **Percent** | **Cumulative Frequency** | **Cumulative Percent** |
| --- | --- | --- | --- | --- |
| **No** | **17245** | **89.56** | **17245** | **89.56** |
| **Yes** | **2011** | **10.44** | **19256** | **100.00** |

**Table S6. Causes of death by transplanted (yes vs no)**

| **Causes of death** | **Transplanted (yes vs no)** | | |
| --- | --- | --- | --- |
|  | **Non-Transplanted** | **Transplanted** | **Total** |
| **Cardiovascular** | 268 | 266 | 534 |
|  | 3.69 | 3.66 | 7.35 |
|  | 50.19 | 49.81 |  |
|  | 7.75 | 6.98 |  |
| **Cerebrovascular** | 42 | 51 | 93 |
|  | 0.58 | 0.7 | 1.28 |
|  | 45.16 | 54.84 |  |
|  | 1.21 | 1.34 |  |
| **Graft Failure** | 3 | 500 | 503 |
|  | 0.04 | 6.88 | 6.92 |
|  | 0.6 | 99.4 |  |
|  | 0.09 | 13.12 |  |
| **Hemorrhage** | 211 | 72 | 283 |
|  | 2.9 | 0.99 | 3.89 |
|  | 74.56 | 25.44 |  |
|  | 6.1 | 1.89 |  |
| **Immunosupression** | 1 | 1 | 2 |
|  | 0.01 | 0.01 | 0.03 |
|  | 50 | 50 |  |
|  | 0.03 | 0.03 |  |
| **Malignancy** | 113 | 906 | 1019 |
|  | 1.55 | 12.46 | 14.02 |
|  | 11.09 | 88.91 |  |
|  | 3.27 | 23.77 |  |
| **Operative** | 1 | 0 | 1 |
|  | 0.01 | 0 | 0.01 |
|  | 100 | 0 |  |
|  | 0.03 | 0 |  |
| **Other** | 2265 | 1322 | 3587 |
|  | 31.16 | 18.19 | 49.35 |
|  | 63.14 | 36.86 |  |
|  | 65.52 | 34.68 |  |
| **Renal Causes** | 16 | 66 | 82 |
|  | 0.22 | 0.91 | 1.13 |
|  | 19.51 | 80.49 |  |
|  | 0.46 | 1.73 |  |
| **Respiratory** | 101 | 165 | 266 |
|  | 1.39 | 2.27 | 3.66 |
|  | 37.97 | 62.03 |  |
|  | 2.92 | 4.33 |  |
| **infection** | 436 | 463 | 899 |
|  | 6 | 6.37 | 12.37 |
|  | 48.5 | 51.5 |  |
|  | 12.61 | 12.15 |  |
| **Total** | 3457 | 3812 | 7269 |
|  | 47.56 | 52.44 | 100 |
| **Frequency Missing = 13210** | | | |
